# Tracking induced pluripotent stem cell differentiation with a fluorescent genetically encoded epigenetic probe

**DOI:** 10.1007/s00018-024-05359-0

**Published:** 2024-09-02

**Authors:** Afanasii I. Stepanov, Alexandra A. Shuvaeva, Lidia V. Putlyaeva, Daniil K. Lukyanov, Adelya A. Galiakberova, Dmitry A. Gorbachev, Dmitry I. Maltsev, Valeriya Pronina, Dmitry V. Dylov, Alexey V. Terskikh, Konstantin A. Lukyanov, Nadya G. Gurskaya

**Affiliations:** 1 Skolkovo Institute of Science and Technology, Bolshoi Blvd. 30, Bld. 1, 121205 Moscow, Russia; 2 Shemyakin-Ovchinnikov Institute of Bioorganic Chemistry, Miklukho-Maklaya 16/10, 117997 Moscow, Russia; 3 Moscow Institute of Physics and Technology, Dolgoprudny, 141701 Russia; 4 Pirogov Russian National Research Medical University, Ostrovityanova 1, 117997 Moscow, Russia; 5 Federal Center of Brain Research and Neurotechnologies, Federal Medical Biological Agency, Moscow, 117997 Russia; 6 The Scintillon Research Institute, 6404 Nancy Ridge Dr., San Diego, CA 92121 USA

**Keywords:** Epigenetics, Histone modification, Fluorescent proteins, Genetically encoded sensor, H3K9me3, Machine learning

## Abstract

Epigenetic modifications (methylation, acetylation, etc.) of core histones play a key role in regulation of gene expression. Thus, the epigenome changes strongly during various biological processes such as cell differentiation and dedifferentiation. Classical methods of analysis of epigenetic modifications such as mass-spectrometry and chromatin immuno-precipitation, work with fixed cells only. Here we present a genetically encoded fluorescent probe, MPP8-Green, for detecting H3K9me3, a histone modification associated with inactive chromatin. This probe, based on the chromodomain of MPP8, allows for visualization of H3K9me3 epigenetic landscapes in single living cells. We used this probe to track changes in H3K9me3 landscapes during the differentiation of induced pluripotent stem cells (iPSCs) into induced neurons. Our findings revealed two major waves of global H3K9me3 reorganization during 4-day differentiation, namely on the first and third days, whereas nearly no changes occurred on the second and fourth days. The proposed method LiveMIEL (Live-cell Microscopic Imaging of Epigenetic Landscapes), which combines genetically encoded epigenetic probes and machine learning approaches, enables classification of multiparametric epigenetic signatures of single cells during stem cell differentiation and potentially in other biological models.

**Supplementary Information:**

The online version contains supplementary material available at 10.1007/s00018-024-05359-0.

## Introduction

The epigenetic state of a cell, which is determined by DNA and histone modifications, plays a crucial role in defining the organization of chromatin. Epigenetic modifications of chromatin are essential for regulating gene expression. Among these modifications, post-translational modifications of histones, such as methylation, acetylation, phosphorylation, etc., can occur at different amino acid positions. The histone modification known as H3K9me3 is not only responsible for suppressing repetitive elements in the genome, but it also plays a significant role in maintaining cell identity [1]. Monoclonal antibodies that specifically target histone modifications have been an invaluable tool in studying the functional significance of these modifications through techniques like ChIP-seq [2]. To overcome the size limitation posed by antibodies, smaller modified versions have been developed, enabling their passage through the nuclear pore and facilitating the visualization of histone modifications in live cells [3–5]. Recent advancements have focused on investigating chromatin at the single-cell level, utilizing high-throughput DNA sequencing to analyze DNA methylation, histone modifications, DNA accessibility, and chromatin conformation [6, 7]. Protein reader domains that specifically bind to modified histones (histone modification reader domains, HMRDs) play a key role in the interpretation of the epigenetic code; HMRDs attract proteins with specific activities required at a given chromatin locus [8]. The concept and implementation of reader domain-based techniques was studied in many laboratories. In a proof-of-concept study published in 2014, the authors tested a specificity of several recombinant histone modification-interacting domains (HMIDs) with ENCODE-validated antibodies [9]. Then, HiMIDs were developed, which are modified HMRDs with double specificity to H3K9me3-H3K36me2/3 using MPP8 chromodomain and DNMT3A PWWP domain, respectively [10]. After that, some HMRD-based sensors with fluorescent proteins were created: cMAPs (H3K4me3-H3K27me3 bivalent probe) [11], heterodimeric sensor for recognizing H3K9me3 [12], eCRs (histone tri-methylation at H3K4, H3K9 and H3K27) [13]. Also, the implementation of reader domain-based approach can detect dynamic epigenetic changes in individual cells and give insights into the basic problems (cell differentiation, neoplastic differentiation or the analysis of chronological versus biological epigenetic clock) as well as to be applied for high-throughput screening.

Human induced pluripotent stem cells (iPSCs) were first obtained in 2007 [14]. Since iPSCs can be obtained from readily available somatic cells of patients, their use solves important methodological and ethical problems, for example, the availability of neuronal cells. There are two main methods for differentiating induced pluripotent stem cells (iPSCs) into neurons [15]. The first method, known as directed differentiation, aims to mimic neurogenesis by adding small molecules and growth factors in order to guide the iPSCs into various stages of neuronal progenitors and different types of neurons. The inhibition of bone morphogenic protein (BMP) and transforming growth factor β (TGF-β) signal pathways, dual SMAD inhibition method, is a widely used traditional approach to generate induced neurons from iPSC [16]. The second method, neuronal induction, involves inducing the expression of specific transcription factors that directly drive the iPSCs into different neuronal types, bypassing the intermediate stages of neuronal progenitor cells [15]. In recent years, the transcription factor NGN2 has gained popularity as a key factor for induced differentiation [17] and showed remarkably short differentiation periods, ranging from a few weeks [18] to just two weeks [19] or even four days [20]. ATOH1 is another transcription factor that has been shown to participate in the development of inner ear hair cells [21], and was utilized in combination with other neurogenesis signals to induce neuronal differentiation in stem cells [22]. Finally, Ng et al. found ATOH1 alone was sufficient to drive differentiation in three different human induced pluripotent stem cell lines [23]. One crucial factor in utilizing iPSCs is selecting an efficient directed differentiation method. Being able to identify alterations in the epigenetic profile throughout the reprogramming process could serve as a valuable supplementary tool for evaluating the effectiveness of the procedure chosen.

Recently, a new platform for high-throughput analysis of epigenetic landscapes in single cells—Microscopic Imaging of Epigenetic Landscapes (MIEL) was published [24]. Using multivariate image analysis and machine learning, it becomes possible to identify hundreds of features of intranuclear distribution of epigenetic marks and compare various cell samples in a virtual multidimensional space through multidimensional scaling (MDS). MIEL is capable of identifying epigenetic landscapes characteristic of various cell lines, detecting drug-induced changes in chromatin, and monitoring chromatin changes during cell differentiation [24, 25]. However, the main limitation of the method is its restriction to fixed cells, which does not allow for the observation of the dynamics of epigenetic changes at the single-cell level.

**Fig. 1:**
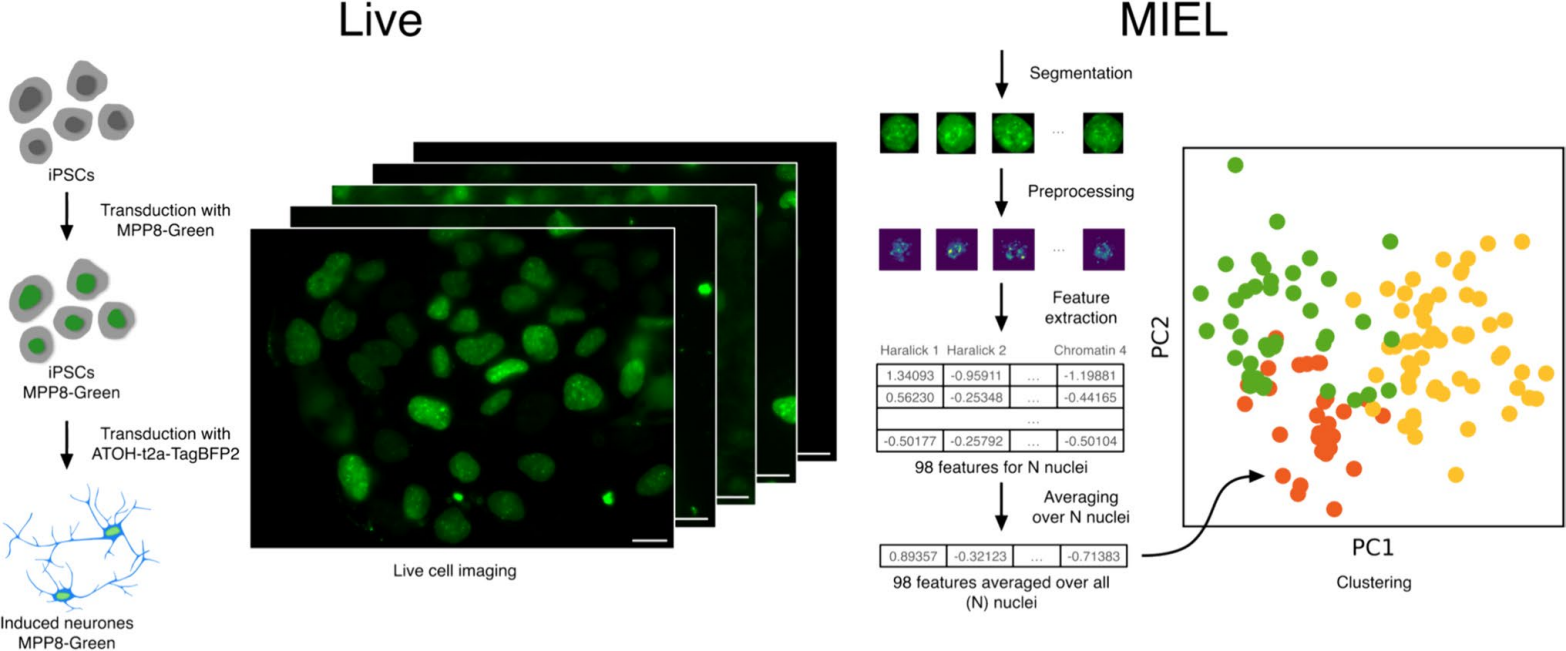
Schematic overview of LiveMIEL. Left: Visualization of living iPSCs expressing the epigenetic sensor MPP8-Green during ATOH1-induced differentiation into induced neurons. Right: The obtained images undergo segmentation, feature extraction for intranuclear epigenetic patterns, and patterns data classification

Here we present LiveMIEL—a combination of live imaging of epigenetic landscapes using an HMRD-based genetically encoded epigenetic probe (GEEP) with MIEL analysis. LiveMIEL was applied to monitor changes in the H3K9me3 landscape during differentiation of iPSCs into induced neurons (Fig. 1).

## Results

### Development of a genetically encoded fluorescent probe for H3K9me3

The first step of our work was to construct a fluorescent genetically encoded epigenetic probe (GEEP) for visualization of epigenetic landscapes in nuclei of live cells. We focused on the histone H3 methylation at lysine 9 (H3K9me3) modification, characteristic for transcriptionally inactive regions of chromatin (heterochromatin) [26]. As a H3K9me3-binding domain, we selected the N-terminal chromodomain (residues 55–117) of human M phase phosphoprotein 8 (for simplicity, we herein refer to this chromodomain as MPP8 throughout the text). MPP8 interaction with the H3K9me3 has been proven by X-ray crystallography [27] and a yeast three-hybrid approach [28]. Importantly, ChIP-seq analysis showed that MPP8 enriched regions correspond to H3K9me3 in human cells and embryonic stem cells [29–31], which makes MPP8 a promising recognizing unit for H3K9me3 visualization. As a fluorescent tag, we used bright monomeric green fluorescent protein mNeonGreen [32]. Also, nuclear localization signal (NLS) was introduced to facilitate accumulation of the probe in the nuclei.

**Fig. 2:**
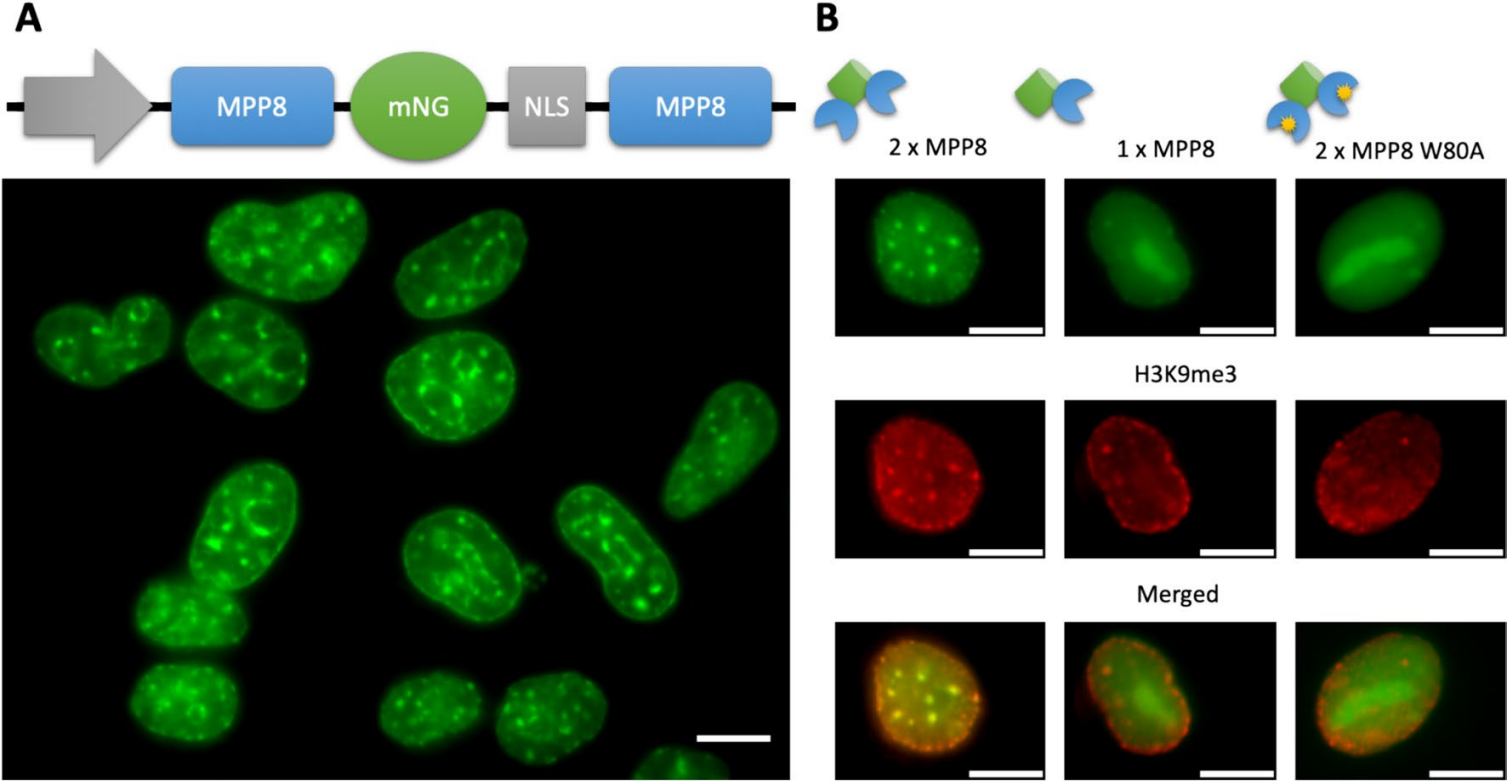
Visualization of H3K9me3 landscapes in living cells with MPP8-based GEEP. **A** Upper panel: schematic representation of MPP8-Green construct; bottom panel: fluorescence microscopy of HEK293T cells transfected with MPP8-Green. **B** Co-staining of MPP8-based constructs with H3K9me3-specific antibodies. Upper panel: schematics; bottom panels: fluorescence microscopy of representative HEK293T cells transfected with a corresponding construct (left, MPP8-Green; middle, MPP8-mNG-NLS; right, MPP8-Green with a binding-inhibiting mutation W80A in both MPP8 domains). Upper photos—green channel for mNeonGreen fluorescence. Middle photos—the same cells in the red channel for H3K9me3 antibody. Bottom photos—merge for the green and red channels. Pearson's correlation values (R) for 2 × MPP8, 1 × MPP8, and 2 × MPP8 W80A are 0.81, 0.16, and −0.07, respectively. Scale bars, 10 $\mu$ m

We designed different variants of the MPP8-based GEEP and tested it by fluorescence microscopy of transiently transfected HEK293T cells (Fig. 2).

We observed no intranuclear patterns for a variant carrying a single copy of MPP8 (MPP8-mNeonGreen-NLS). At the same time, a variant with two MPP8 copies (MPP8-mNeonGreen-NLS-MPP8) produced clear fluorescent patterns (Fig. 2A). These results are fully in line with previous data on other reader domains, where only two (but not one) HMRD copies ensured specific fluorescent patterns [11–13]. The correctness of MPP8-mNeonGreen-NLS-MPP8 patterns was confirmed by co-immunostaining with anti-H3K9me3 antibody, which showed strong correlations (Fig. 2B). In addition, we tested a W80A mutation, which is known to eliminate specific binding of MPP8 to H3K9me3[33]. As expected, the construct with two mutated MPP8 copies (MPP8-W80A-mNeonGreen-NLS-MPP8-W80A) showed a uniform intranuclear distribution with no specific patterns (Fig. 2B). Thus, we concluded that the new GEEP MPP8-mNeonGreen-NLS-MPP8, hereinafter referred to as MPP8-Green, works properly to enable visualization of H3K9me3 distribution patterns in live cells.

**Fig. 3:**
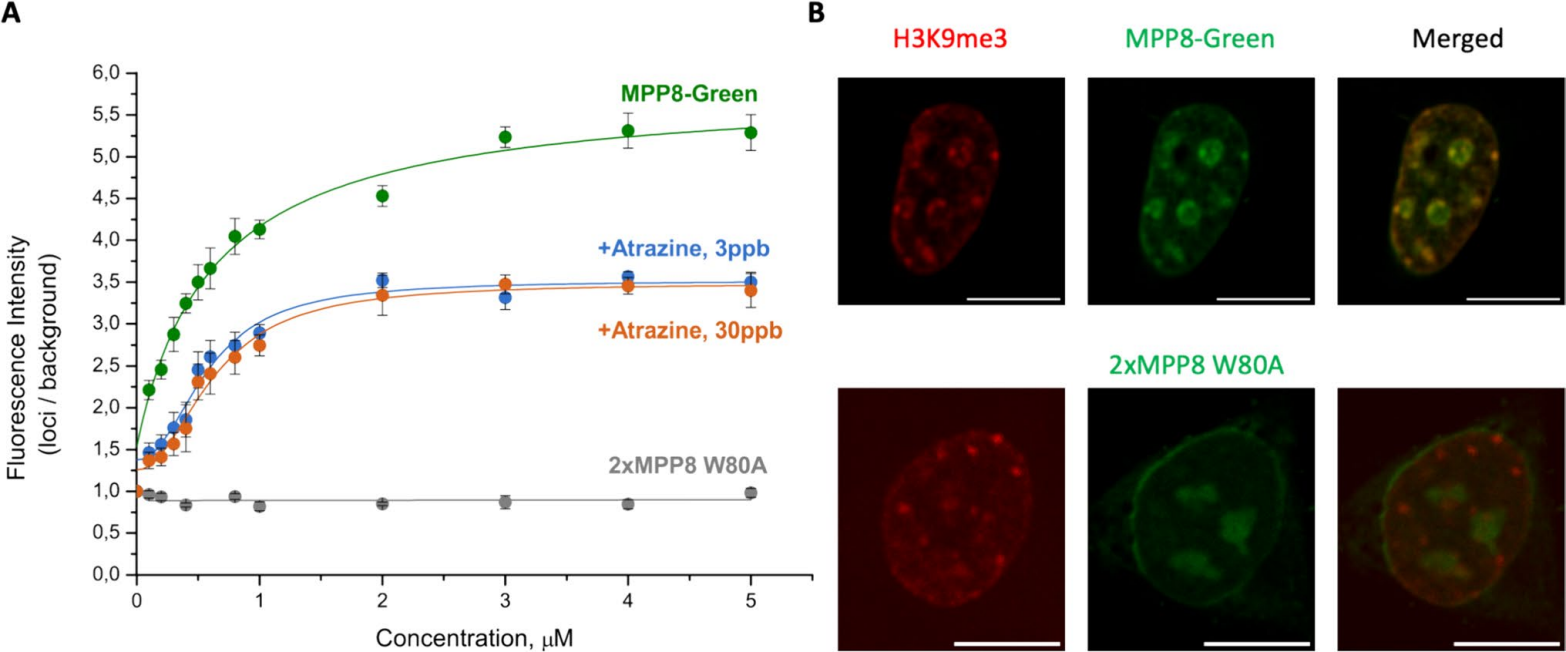
Binding of the MPP8-Green to H3K9me3 in fixed HEK293 cells. **A** Graph showing the dependence of the ratio of fluorescence intensity of loci to background on the concentration of MPP8-Green. Green—MPP8-Green, blue—MPP8-Green after treatment with atrazine 3 ppb, orange—atrazine 30 ppb, gray—2xMPP8-W80A. The curve was fitted using the Hill1 function. Error bars denote SD; **B** Co-staining of HEK293 cells with anti-H3K9me3 antibody and purified MPP8-Green sensor. Pearson's correlation values (R) for MPP8-Green and 2xMPP8 W80A are 0.82 and 0.37, respectively Scale bars, 10 $\mu$ m

Using the CelluSpots histone peptide arrays method, it was demonstrated that MPP8 exhibits specific and robust binding affinity to H3K9me3, along with weaker binding affinities to H3K9me2 and H3K27me3 [9, 34]. This highlights the domain’s selectivity for the H3K9me3 modification. To further evaluate the affinity of MPP8-Green for H3K9me3, we conducted the in situ experiment to assess its binding to histone modification. Initially, we cloned our sensor into a bacterial vector under the control of the T5 promoter, and added a His-Tag for subsequent protein isolation and purification. Following successful protein isolation, the sensor was titrated in fixed HEK293 cells to determine its specific binding to the H3K9me3. Titration was performed across a concentration range from 0.1 µM to 8 µM. Analysis of the resulting images allowed for the calculation of the Kd, which was determined to be 0.49 μM using the Hill1 function, indicating the significant affinity to H3K9me3 (Fig. 3A).

In addition to the MPP8-Green sensor, a similar experiment was conducted with a sensor variant containing the 2xMPP8 W80A mutation, leading to the loss of specific binding to H3K9me3. This variant failed to exhibit any binding to H3K9me3, further confirming the specificity of our sensor. Furthermore, fixed cells were stained with an anti-H3K9me3 antibody and subsequently with the isolated MPP8-Green sensors and 2xMPP8 W80A mutation sensor variants, indicating the MPP8-Green specificity for H3K9me3 (Fig. 3B). Moreover, through an in situ test, the sensor’s sensitivity towards changes in H3K9me3 levels was demonstrated. Previous studies using another H3K9me3-specific sensor revealed that atrazine can reduce the overall levels of H3K9me3 [12]. Therefore, we opted to utilize this chemical compound to induce alterations in H3K9me3 levels. Pre-treatment of HEK293 cells with atrazine one day before the experiment, followed by sensor titration, revealed the sensor's sensitivity to changes in H3K9me3 levels, underscoring its potential for monitoring epigenetic modifications effectively **(**Fig. 3A**)**.

### Visualization of H3K9me3 patterns during iPSC differentiation

Next, we applied MPP8-Green to track changes of H3K9me3 landscapes during iPSC differentiation. Classical methods of iPSCs differentiation take a long time (weeks) and specialized conditions [16]. In contrast, recently developed methods based on overexpression of specific transcription factors ensure fast iPSC differentiation in common media [20, 23]. In particular, overexpression of the transcription factor ATOH1 was found to result in fast (4 days) and very efficient differentiation of iPSCs into induced neurons [23]. Therefore, we chose ATOH1-induced iPSC differentiation as a model to demonstrate the utility of the MPP8-Green sensor to track epigenetic changes during cell differentiation.

**Fig. 4:**
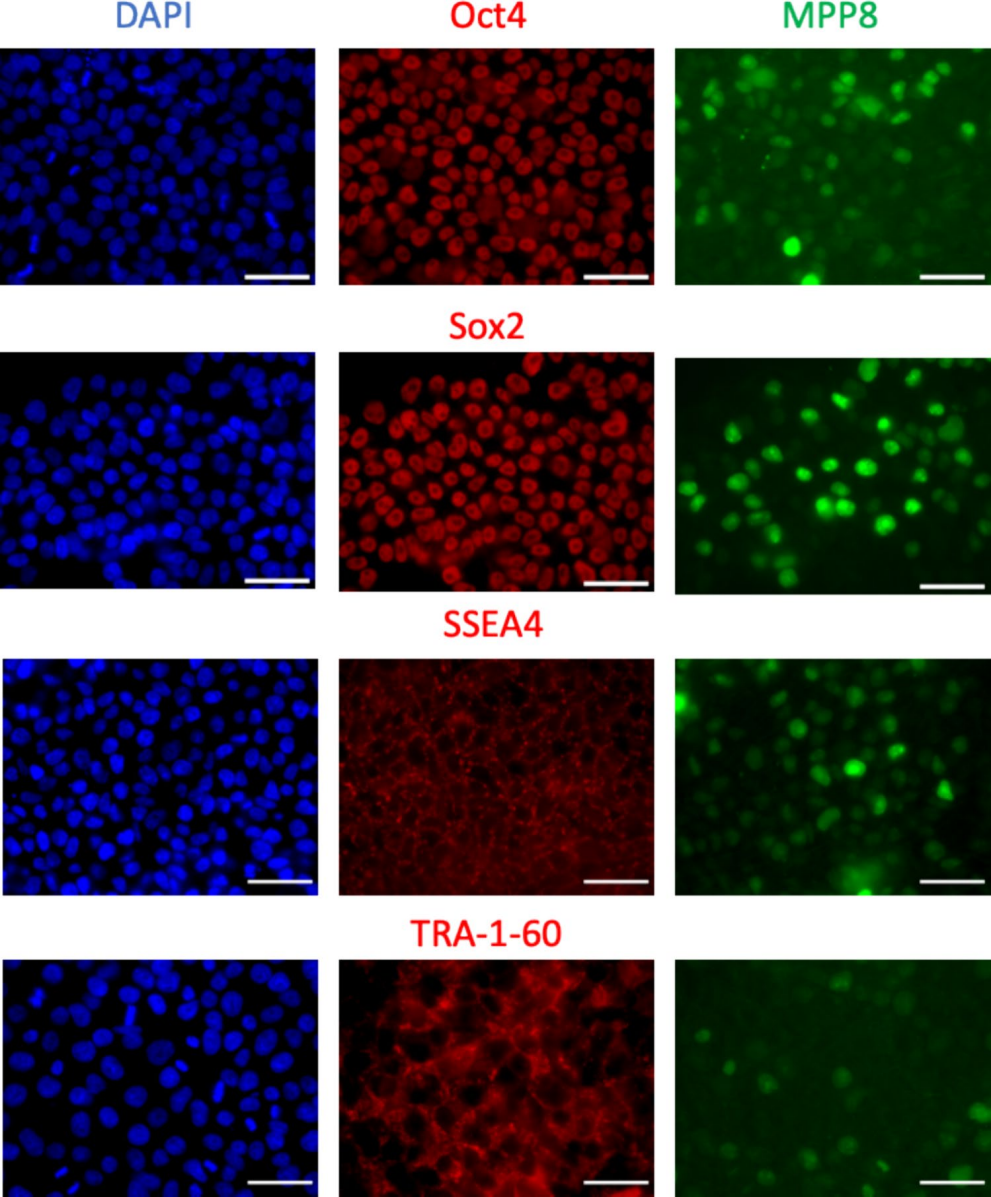
Verification of the main pluripotency factors' expression in iPSC-MPP8-Green cell line. Fluorescence microscopy of fixed cells stained with DAPI (blue channel) and specific antibodies for Oct4, Sox2, SSEA4, and TRA-1–60 (red channel). Fluorescence of the MPP8-Green sensor in the green channel is also shown. Scale bars, 50 $\mu$ m

First, iPS cell line stably expressing MPP8-Green was created by lentiviral transduction and subsequent cell sorting. Immunostaining was carried out for the pluripotency factors Oct4, Sox2, SSEA4, and TRA 1–60, which demonstrated that the pluripotent state of iPSCs was not affected by the overexpression of the probe (Fig. 4).

**Fig. 5:**
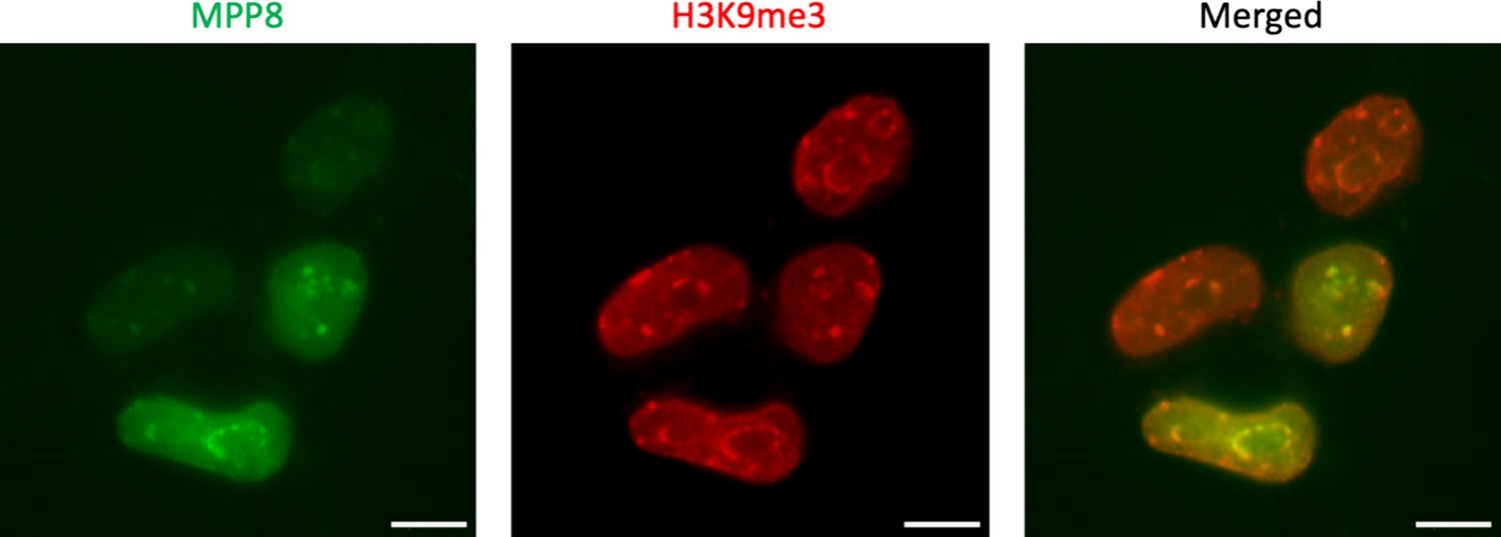
Immunostaining of iPSC-MPP8-Green cells with anti-H3K9me3 antibodies. The first image shows four cells with different levels of mNeonGreen signal. The second image shows anti-H3K9me3 antibody staining. The third image displays the overlay of the two images, demonstrating consistent correlation between the sensor with different signal levels and the antibody. Pearson’s correlation coefficient R = 0.924 for bright nuclei and R = 0.920 for nuclei with low fluorescence intensity. Scale bars, 10 $\mu$ m

After infecting iPSCs with MPP8-Green lentivirus sensor and sorting only green positive cells, we observed heterogeneity in the mNeonGreen signal (Fig. 5).

Some cells exhibited clear enhanced expression of the sensor, while others showed a weak signal. Immunostaining of iPSC-MPP8-Green cells with anti-H3K9me3 antibodies showed that the corresponding patterns are independent of the sensor expression level. In both cases, there was a significant correlation with the antibodies (Fig. 5). Nevertheless, in subsequent analyses, we selected cells with clearly distinguishable patterns regardless of the signal level.

**Fig. 6:**
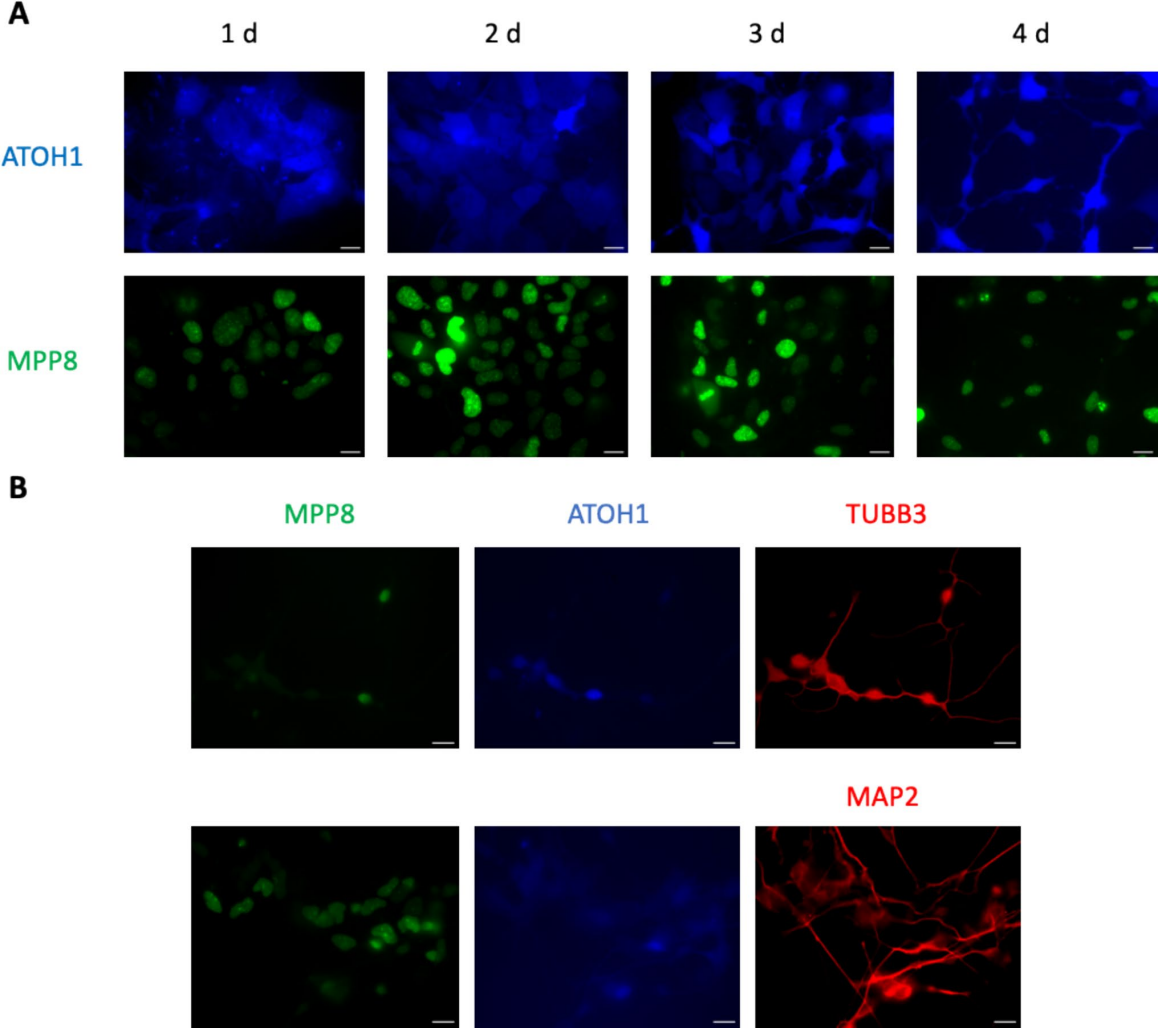
Morphological changes in iPSC-MPP8-Green stable cell line following lentiviral transduction with ATOH1-t2a-TagBFP2. **A** Microscopy of IPS cells during the four days post-transduction reveals changes in cell morphology and H3K9me3 histone modification. Blue channel—fluorescence of TagBFP2 from ATOH1-t2a-TagBFP2 construct; green channel—MPP8-Green. **B** Antibody staining demonstrating the neuronal state of ATOH1-induced cells. Green channel—MPP8-Green; blue channel—ATOH1-t2a-TagBFP2; red channel—antibody staining against TUBB3 (top) or MAP2 (bottom). Scale bars in A and B, 20 $\mu$ m

We constructed a lentiviral vector the ATOH1 gene fused to TagBFP2 via a "self-cleaving" t2a peptide under the constitutive PGK promoter. Stable iPSC-MPP8-Green line was transduced with ATOH1-t2a-TagBFP2 lentivirus. We performed live cell microscopy in two channels: blue channel to select ATOH1-t2a-TagBFP2-expressing cells, and green channel to follow H3K9me3 changes using MPP8-Green (Fig. 6A).

TagBFP2 signal was detected in 24 h with further signal increase, which confirmed the expression of ATOH1. Four days after transduction, the cells acquired a neuronal morphology as seen in the blue channel. The neuronal morphology of the ATOH1-induced cells was confirmed by immunostaining with anti-TUBB3 and anti-MAP2 antibodies (Fig. 6B). We concluded that iPSC-MPP8-Green successfully underwent ATOH1-induced differentiation into induced neurons.

### Transcriptome analysis of iPSC MPP8-Green and WT samples

**Fig. 7:**
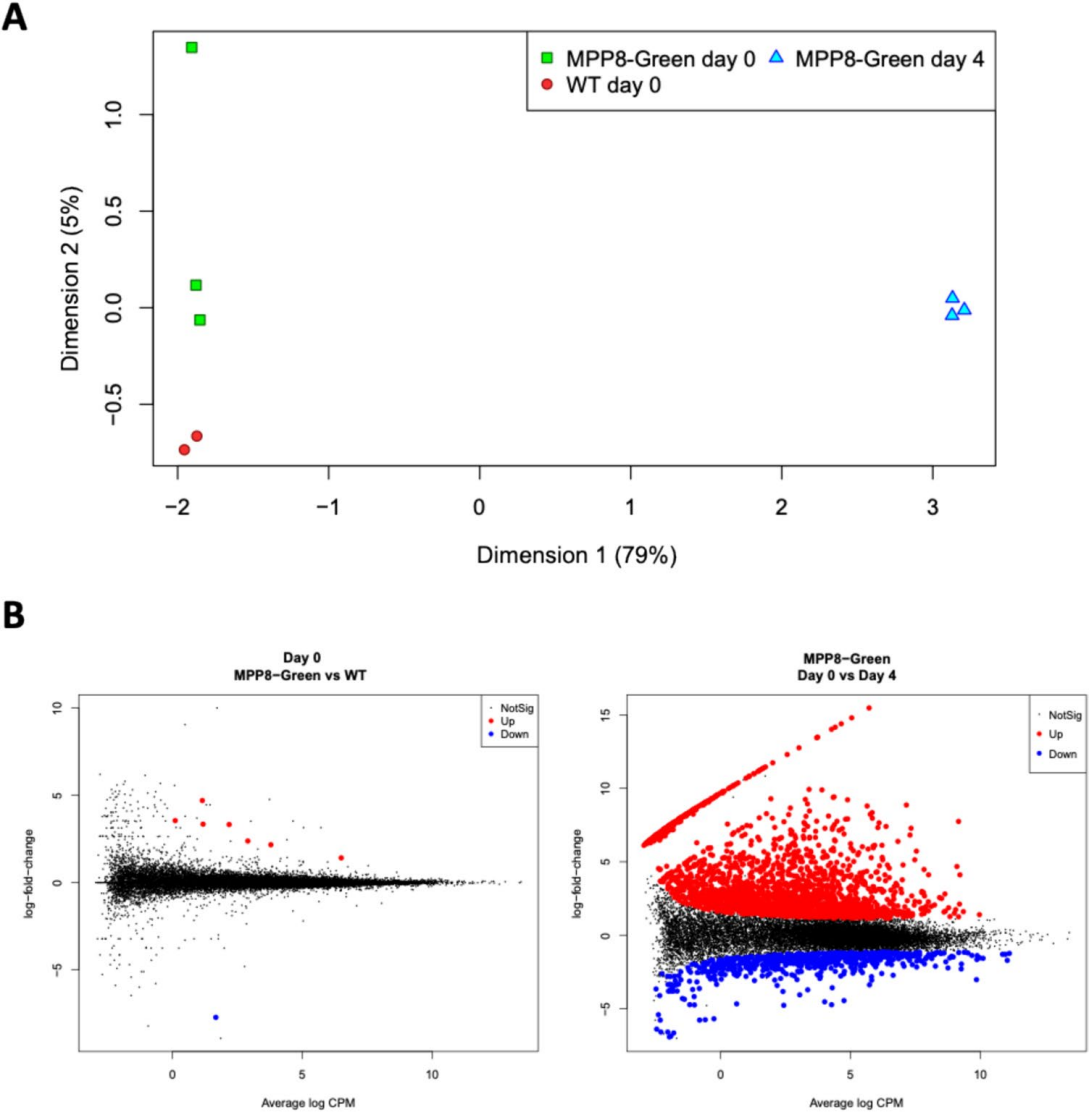
Multidimensional scaling plot and differential expression analysis of iPSC MPP8-Green and WT samples. **A** Multidimensional scaling plot of the samples. Distances between samples approximate the pairwise expression changes between each pair of samples. X and Y axis correspond to the dimensions (eigenvectors) that explain the greatest percentage of total expression variance, see Methods. **B** Mean-difference plots showing average expression (log counts-per-million, logCPM) against logFC for the day 0 MPP8-Green vs WT samples comparison (left) and day 0 vs day 4 MPP8-Green comparison (right) for each gene. Differentially expressed genes with fold-change greater than 2 are highlighted

To estimate the potential impact of GEEP constant expression on iPCS physiology, we performed RNA-seq analysis. MPP8-Green and WT day 0 samples were analyzed to assess the transcriptome similarity of iPSC, while MPP8-Green day 0 and day 4 samples were compared to evaluate whether the process of differentiation has occurred. Multidimensional scaling plot, in which distances correspond to calculated pairwise expression differences between samples, showed that the distances between day 0 and day 4 samples are several times larger than those between day 0 MPP8 and WT samples (Fig. 7A).

Indeed, differential expression comparison between MPP8-Green and WT day 0 samples revealed only 8 significant genes with fold changes significantly greater than 2, while about 3000 genes exhibited such fold changes in the analysis between day 0 and 4 samples (Fig. 7B, Supplementary Table 1).

**Fig. 8:**
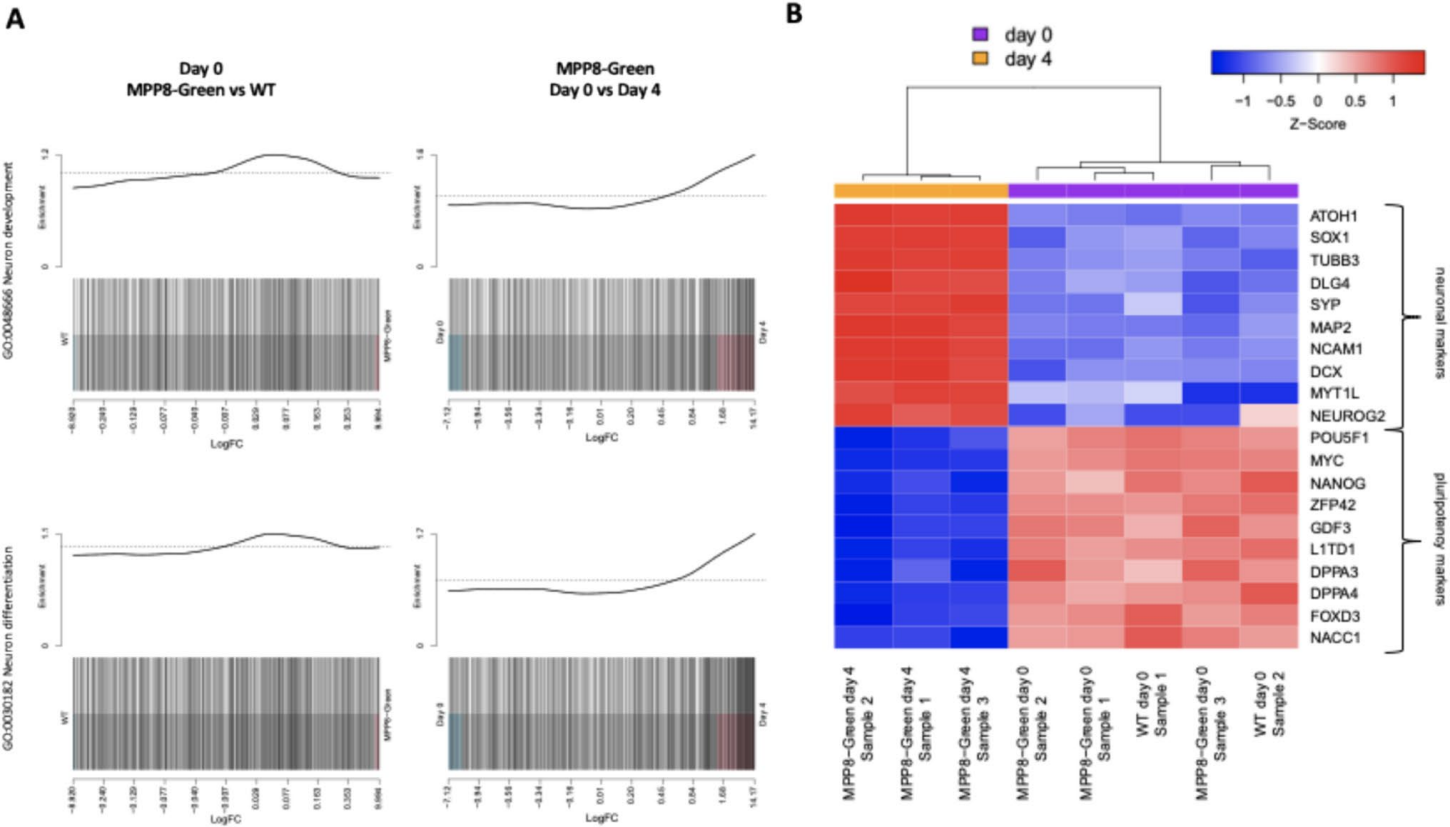
Gene set enrichment analysis and heatmap of iPSC MPP8-Green and WT RNA-seq samples. **A** Barcode plots showing the enrichment of Neuron Development (top) and Neuron Differentiation (bottom) GO terms in the differential expression for the day 0 MPP8-Green vs WT samples (left) and day 4 vs day 0 MPP8-Green comparison (right). The genes are ranked by logFC on the X axis, vertical bars highlight the genes falling into the GO category. The upper panel shows the relative enrichment of the vertical bars in each position. **B** Hierarchical clustering of scaled gene expression profiles (logCPM) for neuron differentiation and pluripotency markers

While differential expression test for any fold change found about 180 differentially expressed (DE) genes between day 0 MPP8 and WT samples, the Gene Ontology analysis did not find any enriched terms associated with neuronal or developmental biology. On the other hand, multiple neuronal Gene Ontology (GO) terms were upregulated for the DE genes between day 4 and day 0 samples (Supplementary Table 1). We additionally tested whether the whole gene sets of neuronal differentiation (GO:0030182) and neuron development (GO:0048666) are differentially expressed. The fry test showed that both gene sets are enriched in day 4 as opposed to day 0 samples (Supplementary Table 1), and do not show differential enrichment between day 0 MPP8 and WT samples (Fig. 8A).

The expression of the chosen neuronal differentiation genes is highlighted on the heatmap (Fig. 8B). Several classical pluripotency transcription factors were significantly downregulated at day 4 versus day 0 when testing for differential expression at any fold change, including POU5F1 (Oct3/4), MYC, NANOG, ZFP42 and others[35] (Fig. 8B). The set of less known gene markers, which was previously shown to specifically distinguish iPSC from iPSC-derived products, was also found to be significantly downregulated in samples at day 4 compared to day 0 [36] (Supplementary Table 1). Overall, the RNA sequencing analysis revealed that the MPP8-Green sensor does not significantly influence the transcriptome and is non-toxic to iPSCs. Additionally, iPSCs with the sensor underwent differentiation correctly.

### Changes of H3K9me3 patterns during iPSC differentiation

To follow dynamics of epigenetics changes during cell differentiation, we imaged live iPSC-MPP8-Green cells before and after transduction with ATOH1-t2a-TagBFP2 once a day for 4 days. For each time point, 10–30 randomly selected fields of view were recorded.

**Fig. 9:**
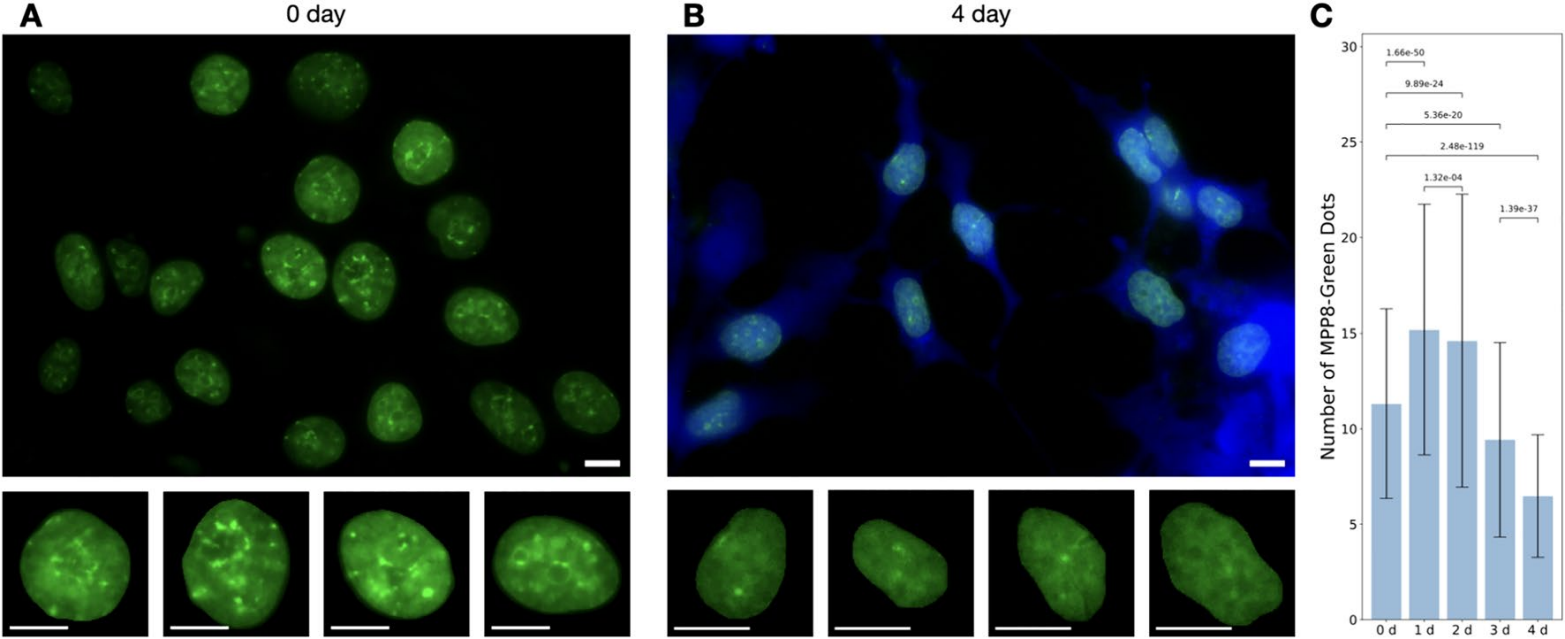
Segmented nuclei from iPSC-MPP8-Green 0 and 4 days of differentiation. **A** Fluorescent microscopy in the green channel (MPP8-Green) of iPSC-MPP8-Green cells on day 0 of differentiation. Segmented nuclei of day 0 differentiation showing a high number of dots and outlining of the nucleoli. **B** Fluorescent microscopy in the green (MPP8-Green) and blue (ATOH1-t2a-TagBFP2) channels of iPSC-MPP8-Green cells on day 4 of differentiation. Segmented nuclei of day 4 differentiation showing 1–2 dots of H3K9me3, while some nuclei lack any discernible patterns and only show diffuse distribution of the sensor. Scale bars, 10 $\mu$ m. **C** Changes of number of MPP8-Green dots per nucleus (a single focal plane images) during ATOH1-induced differentiation. Data = mean ± S.D, 4591 nuclei (800–1000 nuclei per day), Mann–Whitney U-test (two-sided), Benjamini–Hochberg false discovery rate correction

In the collected microscopy images of H3K9me3 changes during differentiation, we observed a clear difference between day 0 and day 4 of differentiation. iPSC-MPP8-Green cells on day 0 showed a higher number of chromatin dots compared to day 4 of differentiation (induced neurons) (Fig. 9).

As seen in the figure, iPSCs on day 0 displayed a higher number of chromatin dots and an outline around the nucleolus, while on the 4th day of differentiation, the number of dots decreased (Fig. 9C), also no nucleolus outlining. At the same time, we did not observe any H3K9me3 differences or global rearrangement in other days of differentiation. However, using our LiveMIEL analysis, we were able to capture time points of H3K9me3 rearrangement that are not visible to the naked eye.

**Fig. 10:**
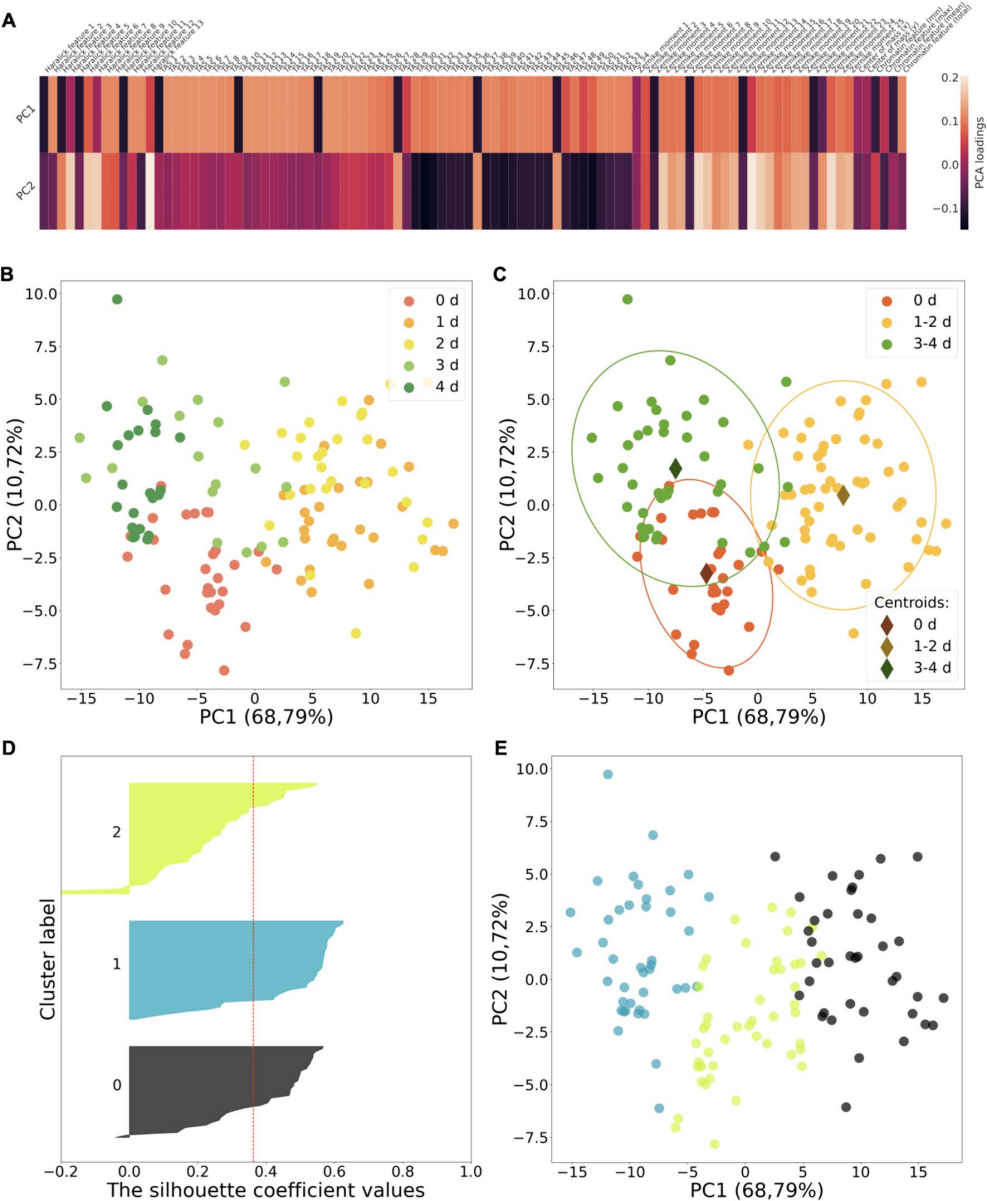
LiveMIEL analysis of H3K9me3 landscapes during ATOH1-induced neuronal differentiation in the iPSC-MPP8-Green cell line. **A** PCA loadings show contribution of the original texture and morphological features to the principal components. **B** Principal component analysis using texture features derived from fluorescent images of MPP8-Green distribution (H3K9me3 patterns) in iPSC nuclei on 0–4 days after ATOH1 transduction. **C** Scatter plot showing 3 groups of H3K9me3 patterns occur during 0–4 days of ATOH1-induced differentiation in iPSC-MPP8-Green cell line. Colored ellipses show 2σ confidence regions for each pattern group. **D** Measuring clustering quality for n = 3 clusters in EM clustering on H3K9me3 patterns data using silhouette coefficient analysis. Silhouette coefficient values *s*_*i*_ $\in [-1, 1]$ for each object in the dataset are shown with colored horizontal bars in the bar chart; red line corresponds to the average value *s* = 0.36 in the dataset; *s*_*i*_ $\approx 1$ indicate that cluster is compact and *i*-th object in cluster is far from any neighboring clusters, *s*_*i*_ $\approx 0$ indicate *i*-th object is on the decision border of neighboring clusters and *s*_*i*_ $< 0$ indicate *i*-th object is closer to objects from another cluster than to the objects in the same cluster. **E** EM clustering on H3K9me3 patterns data for n = 3 clusters. Points represent texture features derived from images of H3K9me3 patterns in nuclei (4 live-cell imaging trials; features values were averaged over N = 40 nuclei). Scatter plots depict the first two principal components for each averaged nucleus

The collected microscopy images of the H3K9me3 patterns were analyzed by the LiveMIEL approach, utilizing principles of the MIEL approach [24]. MIEL uses texture and morphological image features, describing homogeneity, contrast and the presence of organized structures within images (Haralick features), relative arrangement of image structure elements and sub-cellular localization (TAS features) and global measure of mass distribution (image moments) to characterize intranuclear epigenetics patterns. Texture and morphological features (a set of 98 features) derived from nuclei images on 0–4 days of differentiation (data from 4 independent experiments) were normalized with z-score and projected into low-dimensional (2D) space using principal component analysis (PCA). PCA loadings for each feature used show that almost all features contribute to both PC1 and PC2 components; large (positive or negative) loadings indicate that a feature strongly affects the principal component (Fig. 10A).

PCA projection retained 79, 51% of information from the original features data(Fig. 10B). Clustering (K-means, EM algorithm) revealed 3 clusters in the PCA data (Fig. 10D, E) correlated with data labeling at 0 day, 1–2 days, and 3–4 days of cell differentiation (scatter plot depicts labeled PCA data) (Fig. 10C). Assessing clustering tendency in PCA data was completed using Hopkins statistics (H = 0.28). H value close to 0 indicates statistically significant clusters in the data. Number of clusters n = 3 for clustering was determined based on results of silhouette coefficient analysis (Fig. 10D, E). Euclidean distances between cluster centroids showed the 1–2 days H3K9me3 patterns data are the most distant from the 0 day data and 3–4 days data (0 and 1–2 days—12; 1–2 and 3–4 days—16; 0 and 3–4 days—7) (Fig. 10C).

**Fig. 11:**
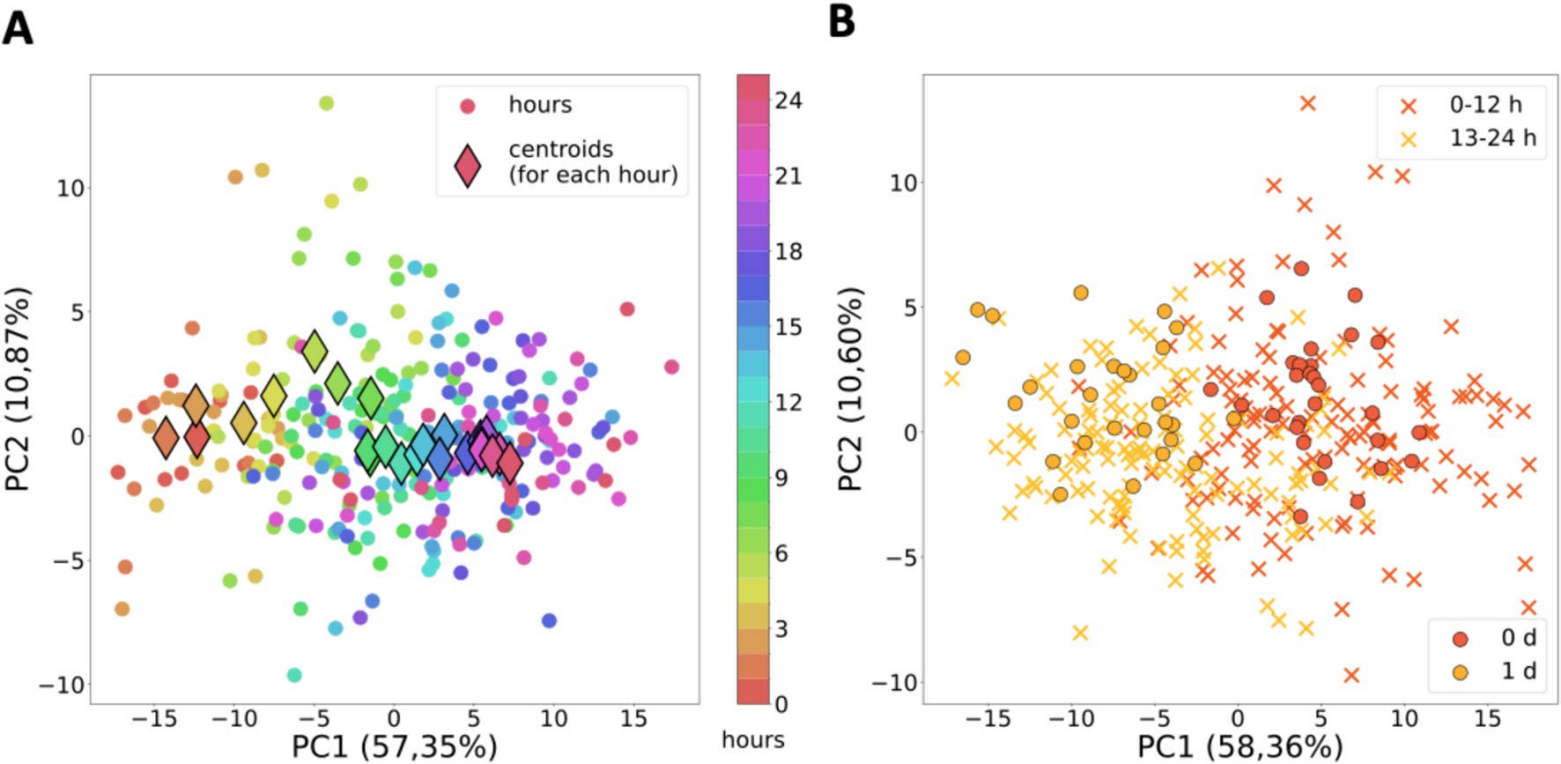
LiveMIEL analysis of H3K9me3 landscapes during 24 h from start of ATOH1-induced neuronal differentiation in the iPSC-MPP8-Green cell line. **A** Principal component analysis using texture features derived from fluorescent images of MPP8-NeonGreen distribution (H3K9me3 patterns) in iPSC nuclei on each hour during 24 h after ATOH1 transduction. **B** Scatter plot comparing H3K9me3 patterns obtained from 24-h and 0–4 days live-cell imaging in the iPSC-MPP8-Green cell line after ATOH1 transduction. Points represent texture features derived from images of H3K9me3 patterns in nuclei: one time-lapse 24-h and four 0–4 days live-cell imaging trials in iPSCs for (**A**) and (**B**) respectively. Features values averaged over N = 20 nuclei for 0–4 h data, N = 30 for 5–17 h data, N = 40 for 18–24 h data and 0–1 days data. Scatter plots depict the first two principal components for each averaged nucleus

The observation of strong epigenetic changes in the early stages of differentiation (1–2 days) prompted us to conduct a detailed investigation of the first day. Therefore, we performed a live imaging experiment over the course of the first 24 h, capturing multiple fields of view at each hour following transduction (Movie 1). The collected images underwent preprocessing and LiveMIEL analysis as described above. Here, the continuous changes in epigenetic patterns were obtained during the first 24 h from start of differentiation (Fig. 11A), most noticeable in the first 8 h with a decreasing rate of changes after the 8th hour. These results were validated by overlaying previous data points from days 0 and 1, serving as references (controls) for 0–12 h and 13–24 h, respectively (Fig. 11B). To investigate which factors have the most significant influence on principal components we calculated PCA loadings as we did it before for 0–4 days of differentiation. Similar to our previous results (Fig. 10A), PCA loadings for 24-h differentiation study (Fig. 11) show that most of TAS, Haralick and Zernike moments features have an impact on both PC1 and PC2 simultaneously. Thus, to perform pattern analysis it appears insufficient to solely choose one or a few parameters, such as visible chromatin dots (Fig. 9). This further justifies and validates our selection of a wide range of texture and morphological features (98) for the purposes of LiveMIEL analysis.

Movie 1**.** Live imaging microscopy of MPP8-Green iPSCs after transduction with ATOH1-t2a-TagBFP2 virus, captured every hour over a 24-h period.

## Discussion

Fluorescent protein-based genetically encoded sensors represent indispensable tools to study diverse processes in live cells in real time [37, 38]. Hundreds of such sensors were constructed for various cellular analytes (e.g., calcium ions) and enzymatic activities (e.g., kinases). Sensors for epigenetic modifications are still an underdeveloped area with a handful of published constructs (reviewed in [39]). Commonly used readouts of genetically encoded sensors are changes in either fluorescence intensity of one FP or Förster Resonance Energy Transfer (FRET) efficiency between two fluorescent proteins [37]. In contrast, LiveMIEL relies on deep image analysis of fluorescent patterns (not intensity) with about hundred features attributed to each nucleus enabling classification of cells according to their epigenetic status. This allows LiveMIEL to provide a novel analysis of the epigenetic landscape, enhancing the information provided by existing genetically encoded probes.

Unlike antibodies, which are derived against synthetic peptides, natural HMRDs recognize histone modifications in context of other modifications of chromatin. Therefore, HMRD-based assessment of epigenome provides a more biologically relevant context-sensitive approach rooted in combinatorial rather than individual pattern of epigenetic marks. The HMRD-based approach will also provide higher reproducibility of analysis. Indeed, batch-to-batch variations is a common problem for antibody-based assays. Stable cell lines engineered with various GEEPs can provide a robust platform to assess epigenetic dynamics in single cells and enable facile implementation of LiveMIEL techniques in different laboratories across the globe. Moreover, imaging without antibodies dramatically lowers the cost of analysis.

A significant concern in live cell imaging with GEEPs is a competition between the probe and endogenous regulatory factors. This problem can be solved by a low expression level of GEEPs. However, this leads to poor image quality and application of strong excitation light, which can be too phototoxic. Also, we recently developed an optogenetic approach enabling on-demand blue light-induced translocation of a GEEP from cytoplasm, where it has no target, to the nucleus [40]. However, it requires a 10–20 min illumination step that complicates multiposition imaging. Thus, we decided to design a probe for the inactive chromatin mark H3K9me3, for which probe-induced artifacts are expected to be negligible even at high expression levels. Indeed, we observed no negative effects of MPP8-Green overexpression in either HEK293T or iPS cells. It was previously demonstrated that the MPP8 chromodomain part (which we used here) does not affect the maintenance of iPSC self-renewal [31]. The MPP8-Green sensor demonstrated specific binding to H3K9me3, enabling visualization of the epigenetic landscape associated with this histone modification. We showed its affinity and sensitivity to changes in H3K9me3 levels using the isolated and purified MPP8-Green protein. Our analysis, conducted in situ on chromatin within fixed cells, provides a context-sensitive measurement unlike previous studies that determine Kd for synthesized H3K9me3 peptides[34, 41]. This context closely mimics the natural environment, offering a more accurate and relevant measure of MPP8’s affinity for H3K9me3. Additionally, unlike traditional antibody staining methods that involve multiple incubation steps and require purchasing antibodies, staining with the MPP8-Green sensor is rapid and immediate upon addition. This efficient technique not only saves time but also ensures swift and precise cell staining, making it an invaluable asset for forthcoming epigenetics research endeavors. MPP8-Green being rapidly expressed and purified from bacterial cells, further simplifying the process compared to purchasing antibodies. It is also worth noting that the limitation and drawbacks, as well as the potential advantage, of our sensor, compared to other probes for H3K9me3, is its context-dependent approach. This means that our sensor loses affinity for H3K9me3 in the presence of phosphorylated S10. This aspect can be viewed from the perspective that our sensor reveals H3K9me3 patterns but remains biologically relevant as it shows H3K9me3 under biological conditions, thereby losing affinity during mitosis due to phosphorylation.

Using the MPP8-Green sensor, we observed two major waves of global H3K9me3 rearrangement during ATOH1-induced human iPSC neuronal differentiation. The first wave occurred within the 1st day after induction, followed by only a subtle change during the 2nd day. This wave is the most pronounced in the initial 8 h, with a decreasing rate of changes after the 8th hour. Then, the cells underwent another major epigenetic shift on the 3rd day, with no significant changes up to the end of observation on the 4th day. The differentiation of ATOH1-induced neurons was published in 2021, so there are no detailed studies on epigenetic changes yet. These results are new in terms of chromatin remodeling during ATOH1-differentiation. LiveMIEL can be used to guide the further ChIP-Seq analysis to particular time points when strong epigenetic changes occur.

Our results are consistent with previous published studies, which have characterized H3K9me3 as a regulator of cellular identity [1]. In particular, this modification has been observed to repress genomic regions responsible for transcription factors out of lineage, effectively blocking the differentiation of iPSCs and maintaining their pluripotency [42, 43].

LiveMIEL appears to be a promising approach that is worth developing further to monitor epigenetic changes in a variety of biological models. In this work, we used a wide-field microscope for LiveMIEL due to its convenience and speed of use. Although this type of microscope may not provide the highest quality images, it still generates sufficient data for the LiveMIEL processing. Therefore, other microscopy systems, such as confocal microscopes, would also be perfectly suitable for this approach. Another potentially competitive advantage of the proposed LiveMIEL approach is small samples required to carry out significant data analysis compared to such techniques as ChIP-Seq. For the reported here study of iPSC differentiation we used datasets consisting of 4500–7500 nuclei and 240–310 nuclei for each analyzed class. Taking into account the averaging we utilized, the minimal number of nuclei required for LiveMIEL in an experiment can be calculated in reverse order from the selected averaging value, but should be not less than 100–200 nuclei on the level of pattern analysis. Ideally, GEEP should be monitored continuously with reasonable time intervals. This would provide a unique opportunity to track epigenetic changes in individual cells and reveal much more details and correlations with cell morphology and/or other live stains, which remain hidden at the ensemble level. A high resolution in time would enable discovery of key stages of the process, which should be then studied in detail using classical methods such as ChIP-Seq. Another direction of improvement is multicolor imaging of two or more GEEPs for different epigenetic marks. This would provide a rich information on spatio-temporal relationship between target histone modifications. Finally, imaging and image analysis of intranuclear epigenetic patterns in three dimensions would strongly enhance the ability of LiveMIEL to classify cells based on 3D architecture of chromatin marks rather than their 2D projections or optical sections.

## Materials and Methods

### Molecular cloning

All DNA constructs were cloned using the Golden Gate cloning system [44]. The genes of interest were cloned into Level 0 and Level 1 backbones obtained from a MoClo Toolkit (AddGene Kit #1000000044). BpiI (BbsI) and Eco31I (BsaI) restriction endonucleases (Thermo Scientific, Waltham, MA, USA) and T4 DNA ligase (Evrogen) were used for the MoClo cloning procedure.

MPP8 reader domain DNA (aa 55–117) was amplified from human placenta cDNA (Takara Bio, USA) with specific primers based on available sequence NM_017520.4 and cloned into Level 0. MPP8-mNeonGreen-NLS-MPP8 and MPP8-W80A-mNeonGreen-NLS-MPP8-W80A were cloned into bacterial vectors under T5 promoter and fused with His-Tag using the Golden Gate cloning system. ATOH1 DNA fragment was amplified from Addgene plasmid pTet-O-ATOH1-T2A-PuroR (Addgene #162342), and put under the control of human Phosphoglycerate Kinase (hPGK) promoter in a lentiviral vector. Transfection efficiency of this plasmid was visualized using mTagBFP2 protein fused to T2A peptide GSGEGRGSLLTCGDVEENPGP. All plasmids were made using a custom backbone based on pRRLSIN.cPPT.EF1 vector, kindly provided by Dr. D. Trono, Lausanne.

### Protein extraction and purification

E. coli cultures were grown in 200 ml of media with 1 mM IPTG induction. Subsequently, the cells were centrifuged using at 4500 rpm for 20 min. The cells were then resuspended in PBS containing 1 mM PMSF and disrupted using an ultrasound Sonicator Q95 (Qsonica, USA). Each sample underwent 20 min of ultrasound treatment. Following sonication, the cell pellets were centrifuged at 4500 rpm for 10 min. His-tagged proteins were purified using TALON metal affinity resin (Takara Bio). A glass column was packed with 1 ml of TALON resin for protein extraction, which was then washed with 10 ml of sterile PBS. The supernatant from the centrifuged tubes was loaded onto the column, and the flow-through was collected. The TALON resin was subsequently washed with 10 ml of 10 mM imidazole (Sigma, Germany) in PBS. The elution of the protein was performed using a 10 ml solution of 250 mM imidazole in PBS. The eluted protein solution was concentrated using an Amicon Ultra-0.5 Centrifugal Filter Unit (Merck, Germany) following the manufacturer's instructions.

### Cell culture and cell line generation

HEK293T cells were cultured at 37 °C (5% CO_2_) in DMEM (PanEco, Moscow, Russia) supplemented with 10% fetal bovine serum (BioSera, Nuaille, France), 100 U/mL penicillin, and 100 mg/mL streptomycin (PanEco). For the live cell imaging experiments, the DMEM was replaced by imaging media: MEM (PanEco) supplemented with 10% fetal bovine serum (BioSera) and 20 mM HEPES (Corning, New York, NY, USA). Initially, a culture of induced pluripotent stem cells (iPSCs), iPS-KYOU, has been obtained at the Shinya Yamanaka laboratory (Kyoto University, Japan) by the reprogramming of adult female skin fibroblasts. The iPS-KYOU cell line was purchased from the ATCC cell bank (KYOU-DXR0109B, ATCC® ACS-1023™). IPSC were cultured in mTeSR (StemCell Technologies, USA) with daily medium changes for optimal growth and Matrigel (Corning, USA) as the surface coating matrix. The stable cell line IPSC MPP8-NeonGreen was registered in the database hPSCreg, the information is available at https://hpscreg.eu/cell-line/KUIFMSi004-A-1.

For transient transfection of HEK293T cells with various GEEP plasmids, PEI 25 K (Polysciences, USA) was used according to the manufacturer's instructions. Stably transduced iPSC MPP8-Green cell line was obtained by lentiviral transduction. Vector particles were generated by PEI 25 K (Polysciences, USA) transient transfection of HEK293T cells. Twenty-four hours before transfection, 1.5 × 10^6^ HEK293T cells were seeded into a 60 mm culture dish. The total of 2 μg and 0.6 μg of the two packaging plasmids pR8.91 and pMD.G, respectively, and 6 μg of MPP8-mNeonGreen-MPP8 or ATOH1-t2a-TagBFP2 were used for transfection. The DNA-PEI mixture was incubated for 20 min at room temperature and then was added dropwise. After 4 h, the medium was replaced with 2 ml of fresh DMEM. Twenty-four hours afterwards the medium containing lentiviral vector particles was filtered (0.45-µm filter) and concentrated by ultracentrifugation at 100,000 g (Beckman, USA) at 4 °C for 3 h. The pellet was resuspended in 500 µl mTeSR (StemCell Technologies, USA) and used for transduction of iPS cells. To create stable cell lines, lentiviral particles were added to 1 × 10^5^ iPSCs or HEK293T. Then transduced cells were sorted with Bio-Rad S3e cell sorter (USA) using mNeonGreen as a selective marker.

### *iPS* cells differentiation

We chose ATOH1-induced differentiation into induced neurons as the model for differentiation due to its simplicity for replication [23]. In contrast to the original differentiation method that utilized tetracycline-induced ATOH1 overexpression, we used constitutive expression of ATOH1 but only added the virus on the day of the experiment. A lentiviral plasmid containing ATOH1-t2a-TagBFP2 was constructed as described above. iPS cells were seeded in confocal dishes with glass bottoms at a density of 0.1 × 10^6^ cells for microscopy. The ATOH1 lentiviruses were produced using a similar protocol as MPP8-mNeonGreen-MPP8. On the day of the experiment, concentrated ATOH1 viruses were added to iPS cells, and after 24 h, the virus-containing medium was replaced with fresh mTesR medium. The medium was changed daily, and the differentiation was monitored for 5 days, starting from before virus addition (as day 0 of differentiation) and up to 4 days after virus addition.

### Live-cell imaging

For the live cell imaging experiments, iPS cells were seeded on glass-bottomed 35 × 10 mm dishes (SPL Life Sciences, Gyeonggi-do, Korea) and were incubated at 37 °C (5% CO_2_) overnight. The next day, the cells were transduced with ATOH1-t2a-TagBFP2 lentiviruses. Imaging experiments were performed using a BZ-9000 inverted fluorescence microscope (Keyence, Osaka, Japan). The resulting images had a resolution of 1360 × 1024 pixels and were obtained using a 2/3-inch camera with 1.5 million pixel monochrome CCD (colorized with LC filter), ultra-high-pressure mercury lamp, 120 W and Nikon CFI 60 Series infinite optical system (for imaging of iPSs differentiation a 60 × PlanApo 1.40 NA oil objective was used). TexasRed OP66838 BZ filter (Keyence, Osaka, Japan) was used to induce red fluorescence, a GFP-BP OP66836 BZ filter (Keyence) was used to induce the mNeonGreen fluorescence, a 49,021-ET-EBFP2/Coumarin/Attenuated DAPI filter (Chroma Technology) was used to induce the TagBFP2 fluorescence. For the live cell imaging experiment conducted over the first 24 h, we obtained images with a resolution of 1024 × 1024 pixels using the Nikon Eclipse Ti2 microscope, equipped with a Filter 525 nm, Nikon’s FX-format F-mount cameras Digital Sight equipped with CMOS image sensors, 60 × Oil CFI Apo TIRF 1,49, WD 0,12.. During imaging, the iPS cells were maintained at 37 °C (5% CO_2_).

### Fixed-cell immunofluorescence

Cells were seeded and grown as described above, fixed in 4% formaldehyde in PBS for 15 min at room temperature, washed three times in PBS, permeabilized for 20 min at room temperature in PBS supplemented with 0.1% Triton X-100 (Helicon, USA), and incubated for 1 h with 1% BSA (Sigma, USA) in PBS for blocking. Corresponding primary antibodies anti-H3K9me3: ab39161 (Active Motif), anti-Oct4: PAA424Hu01 (Cloud-Clone Corp.), anti-Sox2: AF7950 (Affinity), anti-SSEA4: RGK25601 (AntibodySystem), anti-TRA 1–60: Ab16288 (Abcam), anti-tubb3: AF700 (Affinity), and secondary antibodies (Alexa Fluor 488 anti-mouse and 568 anti-rabbit IgGs from ThermoFisher) were diluted in PBS containing 0.02% BSA. Primary antibody incubations were performed 1 h at room temperature and secondary antibody incubations were performed also for 1 h at room temperature. Cells were washed with PBS and imaged in imaging media using a BZ-9000 inverted fluorescence microscope (Keyence, Osaka, Japan). In the titration experiment with sensor methanol was utilized instead of PFA, while keeping the rest of the protocol unchanged. Additionally, Leica TCS SP2 laser scanning confocal microscope with 63 × oil immersion objective was employed for this experiment.

### Colocalization analysis

The colocalization analysis was conducted using the ImageJ platform. Pearson correlation was employed to analyze binding colocalization. Costes' thresholding algorithm was utilized for thresholding.

### RNA sequencing

RNA iPSC wild-type, RNA iPSC-MPP8-Green, and RNA from iPSCs on the 4th day of differentiation were isolated using the RNA Solo kit (Evrogen). The quality of isolated RNA was assessed using the Agilent Bioanalyzer. The isolated RNA was utilized for library preparation with the NebNext UltraII RNA directional kit. The resulting libraries were sequenced in paired-end mode with a read length of 151 base pairs on the Novaseq6000 platform (Illumina). Library preparation and sequencing were conducted at Skoltech’s Genomics Core Facility.

### RNA-seq data analysis

Raw fastq files were mapped to genome (GRCh38) using Hisat2 software with genome_snp_tran index [45]. Stringtie software was used to count features. After obtaining the count table, downstream analysis was done in edgeR [46]. Genes with low expression across samples were filtered using filterByExpr function, and TMM normalization performed. The normalization factors ranged from 0.97 to 1.02, indicating similarity of gene composition between samples. MDS plot was constructed using limma’s plotMDS function with pairwise gene selection. Distances between samples on the MDS plot correspond to the pairwise leading logFC changes between each pair of samples, which is calculated as the root-mean-square average of the top 500 largest logFC changes between them. The common, trended and tagwise dispersions were estimated using estimateDisp with robust = T hyperparameter, with the common dispersion equal to 0.0074. Conventional differential expression test (quasi-likelihood F-test) for day 4 vs day 0 samples produced more than 13,000 significant hits, and about 200 genes for the day 0 MPP8 vs WT samples. To narrow down the list of genes for more biological relevance, we used glmTreat method to test for genes that have fold-changes higher than the specified threshold of 2. Heatmap and complete linkage hierarchical sample clustering was performed using the coolmap function from limma package on logCPM values. GO analysis was performed with goana function. For gene set testing, fry test implemented in fry function of the GO.db package was used and gene set enrichment visualized using barcodeplot function on the logFC statistics from the limma R package[47].

### Image and data processing

#### Live-cell images segmentation

Objects (nuclei, cells) segmentation from fluorescent microscopic images was carried out by step-by-step applying of bandpass filtering, watershed segmentation and procedure of filtering out false-positive objects to the images (custom algorithms). Prior to segmentation, the pixel values of the images were normalized by intensity to [0, 1] and then images passed through noise removal (image smoothing) and background subtraction using Gaussian blur: image convolution with a blur kernel to remove noise (low σ values) and with a blur kernel to subtract background (high σ values). Image areas were considered segmented with pixel values above a specified threshold. Watershed segmentation was used to separate closely spaced objects in the original image mask obtained via bandpass filtering. Segmented objects with a maximum intensity value below μ * k (μ represents the average intensity value of an image pixel with a mask; k is a configurable parameter varied within [1.2, 2.5]) or those with a small image area were identified as false-positives and removed from the analysis.

#### Data selection

Nuclei images of cells expressing both green (MPP8-Green) and blue (ATOH1-t2a-TagBFP2) fluorescence signals were selected for LiveMIEL analysis. Cells were considered received ATOH1-t2a-TagBFP2 construction if they had matching cells images in blue channel, i.e. for which Euclidean distance between nucleus in green channel and cell in blue channel didn’t exceed half of the cell body radius. Euclidean distances were calculated between centers of mass for segmented nuclei and segmented cells in original (unsegmented) green and blue images respectively.

#### Data processing

Fluorescence patterns profiles for each nucleus were represented using a vector of image features. The vector’s length is given by the number of chosen textural and morphological image features (98 per nucleus). Features including Haralick features (13 features), Threshold Adjacency Statistics (TAS) (54 features) and Zernike moments (25 features) were calculated for nuclei images using Mahotas Library [48]. Coordinates of the center of mass of the nucleus image (2 features) and statistics of chromatin distribution including the minimum, maximum, average and total areas of chromatin dots within the nucleus normalized to the nucleus area (4 features) were calculated using a custom algorithm. Segmentation of chromatin dots within the nucleus was done using the same segmentation strategy as for nuclei, cells segmentation from the original images. Raw feature values were normalized by z-scoring to the zero mean and unit variance of all nuclei being compared. To improve clustering, averaged vectors were calculated in which every element is the average value of N cells in that class (day, hour of differentiation from one imaging trial) for a particular feature. The averaging number N was chosen empirically based on the number and quality (blurring, background illumination) of nuclei images, as well as the variability in nuclei sizes and shapes (2D optical sections of 3D objects) in each averaging set. Averaging was chosen to be the minimum necessary to remove outliers and reduce the influence of the shapes and sizes of nuclei on the texture features derived from the nuclei images. Thus, numbers N = 30, 40 for 250–350 nuclei per class (day or hour of differentiation from one imaging trial) were chosen.

#### Dimensionality reduction

Dimensionality reduction of fluorescence patterns data (set of 98-features vectors) was conducted using the Principal Component Analysis (PCA). Assessing PCA quality was done by calculating explained variance ratio (data information retained) for the first 10 principal components. Data from two first principal components PC1 and PC2 was displayed as a 2D scatter plot.

#### Clustering

PCA data clustering was conducted using K-means and EM algorithms (scikit-learn library implementation[49]). Appropriate number of clusters n was determined using silhouette coefficient analysis of PCA data. For this, silhouette coefficients ([-1, 1]) were calculated for each data object, and the average silhouette coefficient was obtained for the entire data set. These values were calculated for clustering results (K-means, EM algorithm) for several estimated values of the number of clusters n ([2; 5]) on PCA data. As the optimal number of clusters n was chosen n corresponding to the high average value of the silhouette coefficient (close to 1) and the presence in each selected cluster of objects with silhouette coefficients above average. Hopkins statistic was calculated to assess a clustering tendency of the data set:

$$H = \frac{{\sum }_{i=1}^{p}{u}_{i}}{{\sum }_{i=1}^{p}{u}_{i} + {\sum }_{i=1}^{p}{w}_{i}}$$

*U*_*i*_ is the distance from the *i*-th object from the data under investigation to the next closest object from the same data (p objects), *w*_*i*_ is the distance from the *i*-th object to the nearest one in a uniform data distribution of p objects. If data is uniformly distributed, H would be close to 0.5. For nonuniformly distributed data H would take values close to 0.

### Supplementary Information

Below is the link to the electronic supplementary material.

Supplementary file1 (XLSX 1384 KB)

Supplementary file4 (AVI 76,805 kb)

## Data Availability

The datasets generated and analyzed during the current study are available from the corresponding author on reasonable request.
